# HDAC1‐mediated deacetylation of LSD1 regulates vascular calcification by promoting autophagy in chronic renal failure

**DOI:** 10.1111/jcmm.15494

**Published:** 2020-06-28

**Authors:** Jiajun Zhou, Han Zhou, Caixin Liu, Lin Huang, Dongmei Lu, Chaoqing Gao

**Affiliations:** ^1^ Kidney Department Yijishan Hospital of Wannan Medical College Wuhu China; ^2^ Queen Mary College of Nanchang University Nanchang China; ^3^ Clinical Laboratory Yijishan Hospital of Wannan Medical College Wuhu China

**Keywords:** autophagy, chronic renal failure, deacetylation, HDAC1, LSD1, SESN2, vascular calcification

## Abstract

Chronic renal failure (CRF) is commonly associated with various adverse consequences including pathological vascular calcification (VC), which represents a significant clinical concern. Existing literature has suggested the involvement of histone deacetylases (HDACs) in the progression of CRF‐induced VC. However, the underlying molecular mechanisms associated with HDACs remain largely unknown. Therefore, we established the adenine‐induced CRF rat model and in vitro VC models based on vascular smooth muscle cells (VSMCs) to examine HDAC1/lysine demethylase 1A (LSD1)/SESN2 as a novel molecular pathway in CRF‐induced VC. Our initial results demonstrated that HDAC1 reduced the formation of VC in vivo and in vitro. HDAC1 was found to deacetylate LSD1, which subsequently led to impaired transcriptional activity in CRF‐induced VC. Moreover, our results illustrated that LSD1 diminished the enrichment of H3K4me2 at the SESN2 promoter. Autophagy was identified as a vasculo‐protective element against calcification in VC. Finally, we found that the inhibitory effects of HDAC1 overexpression on VC were partially abolished via over‐expressed LSD1 in adenine‐induced CRF model rats and in high phosphate‐induced VSMCs. Taken together, these results highlight the crucial role of HDAC1 as an antagonistic factor in the progression of VC in CRF, and also revealed a novel regulatory mechanism by which HDAC1 operates. These findings provide significant insight and a fresh perspective into promising novel treatment strategies by up‐regulating HDAC1 in CRF.

## 1 INTRODUCTION

Chronic renal failure (CRF) is one of the most prevalent clinical burdens to society worldwide, mainly afflicting the elderly population.^1^ CRF is a syndrome characterized by progressive and irreversible deterioration of renal function,^2^ which often arises as a consequence of tubulointerstitial fibrosis manifesting in interstitial expansion with accumulation of extracellular matrix (ECM), inflammation, tubular atrophy and vascular obliteration.^3^ The main contributory factors implicated in the pathogenesis of CRF include hypertension, glomerulonephritis and diabetes mellitus.^4^ When compared to patients with other chronic diseases, CRF patients tend to require longer and more frequent hospitalizations that are often associated with higher rates of morbidity and mortality.^5^ Therefore, there is an urgent need to develop a more specific and efficient preventive strategy against CRF.

Histone deacetylases (HDACs) can induce the deacetylation of histone and non‐histone proteins, which exerts a major role in regulating both physiological and pathological gene expression.^6^ The pharmacological inhibition of HDACs has been widely reported to function as a suppressor against the initiation of renal fibrogenesis in the obstructed kidney along with cyst formation in polycystic kidney disease.^7^ As a member of the HDAC superfamily, HDAC1 plays a key role in renal protection and regeneration of acute kidney injury.^8^ The specific genetic knockdown of histone deacetylase HDAC1 is a prerequisite factor for the suppression of growth and survival of renal carcinoma cells.^9^ The inhibition of HDAC1 activity either through chemical inhibitor or by genetic silencing enhances vascular calcification (VC), which strongly correlates with a variety of cardiovascular and metabolic diseases.^10^ HDAC1 can play a regulatory role in the autophagy that is a crucial component of metabolic homoeostasis of various tissues via the recycling of cellular constituents, especially under conditions of caloric restriction.^11^ Deregulated autophagy promotes autoimmunity and renal damage.^12^ In functional terms, HDAC1 mediates the deacetylation of lysine‐specific demethylase 1 (LSD1) at K374 in the substrate binding lobe of the enzyme, whereby histone 3 binding influences the gene expression activity of LSD1.^13^ Also known as KDM1A, LSD1 was the first identified histone as demethylase, which often has extensive expression in various human malignancies, including gynaecological cancers.^14^ Notably, the inhibition of LSD1 can induce autophagy in several mammalian cell lines by regulating the expression of autophagy‐related genes.^15^ In the current study, we aimed to exploit models in vitro and in vivo to investigate the potential therapeutic application of HDAC1 in CRF, focusing on its capacity to mediate LSD1 in VC, while also seeking to elucidate the associated underlying molecular mechanisms of this phenomenon.

## 2 MATERIALS AND METHODS

### 2.1 Ethics statement

The current study was performed with the approval of the Ethics Committee and Experimental Animal Ethics of Yijishan Hospital of Wannan Medical College (201902042). Extensive efforts were made to minimize animal numbers and their discomfort.

### 2.2 Adenine‐induced CRF rat models

A total of 40 male Sprague‐Dawley rats (aged 5‐7 weeks old and weighing 200‐220 g) purchased from the Experimental Animal Center of the Chinese Academy of Sciences (Shanghai, China) were used in this study. The rats were fed with modified chow containing 0.75% of adenine for first establishment of the CRF model. They then were placed on an additional high‐phosphorus diet (1.3% phosphorus, 1.06% calcium, 1000 IU/kg vitamin D3 and 23% protein) to induce VC. Following the four‐week period of model establishment, 36 rats were randomly selected and assigned to six groups of six animals each: blank group (normal diet), CRF + VC group, CRF + VC +short hairpin negative control (sh‐NC) group, CRF + VC + sh‐LSD1 group, CRF + VC + sh‐Sestrin2 (SESN2) group and CRF + VC + sh‐LSD1 + sh‐SESN2 group. The rats were injected with 4 × 10^8^ pfu shRNA lentivirus vector (prepared by Shanghai GenePharma Co. Ltd.) or 8 μL in vivo transfection reagent (Polyplus‐transfection Inc) mixed in 600 μL of 10% glucose via tail vein,^16^, ^17^ with ongoing access to normal forage. The rats were housed under controlled conditions at 22°C, 50% humidity with a 12‐hour light/dark cycle and granted free access to food and water. At five weeks after transfection, the rats were anaesthetized with 2% pentobarbital sodium (30 mg/kg) for collection of blood via puncture of the abdominal aorta. The collected blood was heparinized and centrifuged, and the plasma was stored at −80°C for later use. Next, the rats were killed, and a 5 mm segment of the thoracic aorta was resected and fixed by immersion in 4% paraformaldehyde for histological observation. The remaining portion of the aorta was used to determine the intravascular calcium content. In addition, the kidneys were resected and fixed by immersion in 10% formalin for histological analysis.

### 2.3 In vitro VC models

Vascular smooth muscle cells (VSMCs) were isolated from rat thoracic aorta. The isolated aorta was then washed with sterilized phosphate‐buffered saline (PBS) and incubated in 1 mL of 0.2% collagenase I solution in Dulbecco's modified Eagle's medium (DMEM) at 37°C for 30 minutes. A longitudinal incision was then made along the collected aortas, and the intima was scraped from the luminal surface. The tissue samples were then cut into small pieces in DMEM (Gibco/Life Technologies) containing 1% penicillin and streptomycin (Gibco/Life Technologies), and incubated in 0.2% collagenase I solution (Gibco/Life Technologies) at 37°C for 30 minutes with gentle shaking. The separated tissues were then added to a culture dish and incubated in DMEM supplemented with 10% foetal bovine serum (FBS) at 37°C with 5% CO_2_ for attachment. To induce VC in VSMCs, the cells were then cultured with a calcification medium (pH = 7.4) containing 2 mmol/L inorganic phosphate (Pi) and 3 mmol/L calcium (Ca^2+^)^18^ for a period of 3‐6 days, with the medium replaced every two days. The first day of culture in the calcification medium was denoted as day 0. On the sixth day, the cells were rinsed twice with PBS, followed by von Kossa staining to determine the calcium deposition.

### 2.4 Calcified nodule formation evaluated by alizarin red S staining

Alizarin red S staining was applied to the extracted thoracic aortas. In brief, the thoracic aortas were collected and stored in 70% ethanol. Each tissue sample was then placed in 10 mL of alizarin working solution supplemented with 0.8% alizarin red S and 0.5% KOH and rotated for 24 hours. The unstained tissues were then removed by rotating for an additional 24 hours in 10 mL of 0.05% KOH. Finally, formation of calcified nodules was analysed under a microscope (Carl Zeiss).

### 2.5 Determination of calcium content

The cells and tissues were decalcified with 0.6 mol/L HCl at 4°C for 24 hours. The calcium content of the HCl supernatant was determined colorimetrically in accordance with the manufacturer's instructions of the Calcium assay kit (QuantiChrom™ Calcium Assay Kit, BioAssay Systems). In brief, 5 μL of the samples was transferred into a 96‐well plate after which absorbance was measured at 570 nm using a microplate ELISA reader (BioTek Instruments). After decalcification, the cells were washed three times with PBS and subsequently solubilized by 0.1 mol/L NaOH and 0.1% SDS. The protein content was determined using a bicinchoninic acid (BCA) Protein Assay kit (Thermo Scientific Pierce). The calcium content of the VSMCs was normalized to the protein content, while that of the tissues was normalized to tissue dry weight.

### 2.6 Evaluation of kidney function

Rat aortic blood samples collected after 12‐14 hours of fasting underwent biochemical analysis. The levels of serum creatinine (SCr), blood urea nitrogen (BUN) and urine protein (U‐pro) collected for 24 hours were measured by turbidimetry using an automated analyser Falcor 300 (Menarini Diagnostics Corp.) in conjunction with a protein array instrument (BKMAM Biotechnology Co., Ltd.).

### 2.7 Von Kossa staining for calcium deposition

Next, to identify calcification of the VSMCs, we conducted von Kossa staining. In brief, the cells were first fixed for 30 minutes with 10% formalin at ambient temperature, rinsed thrice with ddH_2_O and then incubated with 5% silver nitrate solution at ambient temperature for 30 minutes. The cells were then either exposed to ultraviolet light for 2 hours or overnight, until colour development was complete. The silver nitrate solution was discarded, whereupon the cells were washed using ddH_2_O and photographed under a microscope.

### 2.8 Real‐time quantitative polymerase chain reaction (RT‐qPCR)

Total RNA content was extracted from the thoracic aorta tissues or VSMCs via a TRIzol kit (#16096020, Thermo Fisher Scientific Inc). The extracted RNA was subsequently subjected to reverse‐transcribed into complementary DNA (cDNA) using a High‐Capacity cDNA Reverse Transcription Kit (Thermo Fisher Scientific Inc). The reaction conditions were set to 37°C for 15 minutes, 85°C for 5 seconds and 4°C for termination. Next, we undertook PCR amplification of the target gene using the following components: 2 μL of cDNA template, 0.8 μL of upstream and downstream primers, 10 μL of Ultra SYBR Mixture and 20 μL of distilled water. The reaction conditions involved pre‐denaturation at 94°C for 5 minutes, 40 cycles of denaturation at 94°C for 30 seconds, annealing at 54.5°C for 30 seconds, extension at 72°C for 30 seconds and final storage at 72°C for 10 minutes. Glyceraldehyde‐3‐phosphate dehydrogenase (GAPDH) was regarded as the internal reference. The primer sequences were synthesized by TaKaRa Company (Table 1). The fold changes were calculated based on the 2^−ΔΔCt^ relative quantification method.

**Table 1:** Primer sequences for RT‐qPCR

Gene	cDNA ID	Sequences (5′‐3′)
HDAC1	3065	F: ACTAGATAGGGACCAGCGCA
		R: AGCTCCTAAGCAGGCACTTG
LSD1	851215	F: CGCCACGGTCTTATCAACTT
		R: GCCAGAAACACCTGAGCCTA
SESN2	83667	F: AGCTGGAGAAGACGGAAAGC
		R: CCAAACGTGGGGTCCTCTAC
GAPDH	2597	F: CAAGGTCATCCATGACCCATTTG
		R: GTCCACCACCCTGTTGCTGTAG

Abbreviations: F, forward; GAPDH, glyceraldehyde‐3‐phosphate dehydrogenase; HDAC1, histone deacetylase 1; LSD1, lysine‐specific demethylase 1; R, reverse; SESN2, sestrin 2.

### 2.9 Western blot analysis

Total protein was extracted from thoracic aorta tissues or VSMCs using radioimmunoprecipitation assay (RIPA) lysis buffer (Shanghai Beyotime Biotechnology Co. Ltd.). A BCA kit (20201ES76, YEASEN Biotechnology Co., Ltd.) was employed to measure the protein concentration. Protein was subsequently separated using sodium dodecyl sulphate‐polyacrylamide gel electrophoresis followed by transfer onto polyvinylidene fluoride membranes via the wet transfer method. The membrane was subsequently probed overnight at 4°C with the following diluted primary antibodies purchased from Abcam Inc: Runx2 (ab23981, 1:100), α‐SMA (ab32575, 1:2000), HDAC1 (ab19845, 1:100), LC3 II (ab243506, 1:2000), p62 (ab155686, 1:2000), LSD1 (ab37165, 1:100), SESN2 (ab178518, 1:1000), p70S6K (ab32529, 1:5000), P‐rpS6 (ab215214, 1:5000), rpS6 (ab40820, 1:500), glyceraldehyde‐3‐phosphate dehydrogenase (GAPDH) (ab8245, 1:5000) and phosphorylated p70S6K (F48493‐0.08ML, 1:2000, NSJ Bioreagents). The membrane was subsequently washed three times with Tris‐buffered saline containing Tween‐20 and incubated at room temperature with horseradish peroxidase‐labelled secondary goat anti‐rabbit (ab205718, 1:2000, Abcam Inc) for 1 hour. Protein quantification analyses were conducted using ImageJ 1.48u software (National Institutes of Health), with the ratio of the grey value of the target band to β‐actin indicating the relative protein expression.

### 2.10 Cell treatment

The primary VSMCs in the thoracic aorta were collected and treated with 0.25% trypsin and passaged at a ratio of 1:3 for further expansion. The cells were then inoculated into 6‐well plates at a cell density of 3 × 10^5^ cells/well. When the cells reached approximately 70%‐80% of confluence, cells in the logarithmic growth phase were harvested for the subsequent experiments.

Cells in the logarithmic growth phase were suspended at a density of 4 × 10^5^ cells/mL and inoculated into a 6‐well plate. Next, 2 × 10^6^ TU of the corresponding lentivirus Polybrene (H9268, Sigma‐Aldrich Chemical Company) at a final concentration of 8 μg/mL was added into 1 mL of serum‐free medium containing 100 U/mL penicillin and streptomycin. After extensive mixing, the medium was added to the cells for transfection. After 48 hours of transfection, puromycin (P8833, Sigma‐Aldrich Chemical Company) was added to each well to screen out the stably infected cells. The corresponding shRNA sequence was inserted into pLKO.1‐EGFP‐Puro plasmid. The recombinant lentiviral plasmids were co‐transfected with helper plasmids (VSV‐G and Gag/pol) into HEK293T cells (American Type Culture Collection) to produce lentiviral particles (the cells were cultured for about 72 hours). Viral supernatants were collected, filtered at 0.45 μm and ultracentrifuged. Construction of recombinant lentiviral plasmids, packaging and concentration measurements of viruses were undertaken by Shanghai GenePharma Co. Ltd. The VSMCs were subsequently treated with 2 mmol/L Pi (pH = 7.4) calcification medium. The high Pi‐induced VSMCs were then treated with 5 mmol/L autophagic inhibitor 3‐MA (M9281‐100MG, Sigma‐Aldrich Chemical Company), 1 mmol/L the autophagic inducer valproic acid (VPA) (PHR1061‐1G, Sigma‐Aldrich Chemical Company) and 10 μmol/L of the HDAC1 inhibitor SAHA (SML0061‐5MG, Sigma‐Aldrich Chemical Company). Finally, the high Pi‐induced VSMCs were transfected with oe‐HDAC1, sh‐HDAC1, 3‐MA, VPA, oe‐HDAC1 + SAHA, oe‐LSD1, sh‐LSD1, sh‐SESN2 and sh‐LSD1 + sh‐SESN2 in addition to their corresponding controls. The sequences are depicted in Table 2.

**Table 2:** Oligonucleotide sequences for transfection

	Sequences (5′‐3′)
sh‐NC	TTCTCCGAACGTGTCACGTTTCAAGAGAACGTGACACGTTCGGAGAA
sh‐HDAC1	CCGGGATGTTGGAAACTACTATTATCTCGAGATAATAGTAGTTTCCAACATCTTTTTG
sh‐LSD1	UAGUAGUAACAGACUUUCCUC
sh‐SESN2	UUUAUACUAGCAUGAUACCUU

Abbreviations: HDAC1, histone deacetylase 1; LSD1, lysine‐specific demethylase 1; NC, negative control; SESN2, sestrin 2.

### 2.11 Chromatin immunoprecipitation (ChIP)

ChIP assay was performed using an EZ‐Magna ChIP TMA kit (Millipore). In brief, the VSMCs in the exponential phase of growth were cross‐linked for 10 minutes using 1% formaldehyde, followed by reaction with 125 mmol/L glycine at room temperature for 5 minutes. The cells were then washed twice with pre‐cooled PBS and centrifuged at   1006.2 *g* for 5 minutes. The cell pellet was suspended in cell lysate in order to prepare a final concentration of 2 × 10^6^ cells per 200 mL. A mixture of protease inhibitors was added to the cells, followed by centrifugation at 2515.5 *g* for 5 minutes and re‐suspension of the pellet with nuclear separation buffer. The cells were then subjected to ultrasonic treatment to produce 200‐1000 bp chromatin fragments. Next, centrifugation at 14 000 *g* and 4°C for 10 minutes was performed, and the supernatant harvested. A total of 100 mL supernatant (DNA fragments) was added with 900 μL ChIP Dilution Buffer and 20 mL of 50 × PIC, as well as 60 μL of Protein A Agarose/Salmon Sperm DNA and mixed well at 4°C for 1 hour. The mixture was then permitted to stand at 4°C for 10 minutes and then centrifuged at 700 rpm for 1 minute. The supernatant was then collected, of which 20 μL was employed as the input.

The supernatant prepared from experimental groups was incubated with the following antibodies from Abcam Inc: 1 μL HDAC1 (ab7028, 1:5), histone H3 lysine 9 acetylation (H3K9ac) (ab4441, 1:25), LSD1 (ab17721, 1:100) and Histone H3 Lys4 dimethylation (H3K4me2) (ab77766, 1:25), respectively. In the NC group, 1 μL of rabbit monoclonal antibody to IgG (ab172730, Abcam Inc) was added in addition to 60 μL of Protein A Agarose/Salmon Sperm DNA followed by rotation at 4°C for 2 hours. After standing for 10 minutes, the mixture was centrifuged at 700 rpm for 10 minutes. After removal of the supernatant, the pellet was washed sequentially with 1 mL portions of low‐salt buffer, high‐salt buffer, LiCl solution and TE (twice). Each tube was eluted twice using 250 mL ChIP Wash Buffer. De‐cross‐linking was conducted using 20 mL of 5 mol/L NaCl. After recovery of the DNA fragments, the promoters of LSD1 and SESN2 in the complex were quantified using RT‐qPCR.

### 2.12 Immunofluorescence staining

The VSMCs were cultured in a culture dish with cover glasses placed on top. When cell confluence reached 50%, the cover glass was removed. The cells were then rinsed three times using PBS and fixed in 4% paraformaldehyde for 30 minutes at room temperature. After 15 minutes of permeation using 2% Triton X‐100, the cells were sealed for 45 minutes using 2% BSA. The sealing solution was then discarded, whereupon the cells were subjected to overnight incubation at 4°C with LC3 II antibody (ab63817, 1:100, Abcam Inc). After three PBS washes, the cells were re‐probed with secondary goat anti‐rabbit IgG H&L (ab150080, 1:400, Abcam Inc) at room temperature for 2 hours. Then 4′, 6‐diamidino‐2‐phenylindole DAPI (2 μg/mL) was added for cell staining followed by mounting on glass slides. The expression of LC3 II was then detected under a fluorescence microscope, and the ImageJ software was used to quantify the fluorescence intensity.

### 2.13 Statistical analysis

Statistical analysis was performed using SPSS 21.0 software (IBM Corp.). All measurement data were expressed as mean ± standard deviation (SD). Data following a normal distribution and with homogeneity of variance between two groups were compared using an unpaired *t* test. Data among multiple groups were compared by one‐way analysis of variance (ANOVA) with Tukey's post hoc test. Any *P* < .05 was considered to indicate a statistically significant difference.

## 3 RESULTS

### 3.1 Down‐regulation of HDAC1 was associated with the formation of VC in vivo and in vitro

**Figure 1:**
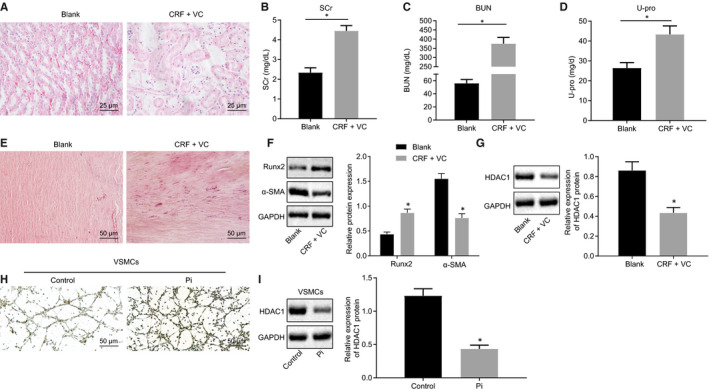
Down‐regulated HDAC1 is associated with the formation of VC in adenine‐induced CRF rats and high phosphate‐induced VSMCs. A, HE staining of renal tissues (400×). B‐D, Serum levels of SCr, BUN and U‐pro measured by the Falcor 300 analyser. E, Calcification of thoracic aorta determined by Alizarin red staining (200×). F, Protein levels of Runx2 and α‐SMA proteins in aortic tissues determined by Western blot analysis. G, Protein level of HDAC1 protein in aortic tissues determined by Western blot analysis. H, Calcium deposition in VSMCs (200×) determined by Von Kossa staining. I, Protein level of HDAC1 protein in VSMCs induced by high Pi determined by Western blot analysis. BUN, blood urea nitrogen; CRF, chronic renal failure; GAPDH, glyceraldehyde‐3‐phosphate dehydrogenase; HDAC1, histone deacetylase 1; HE, haematoxylin‐eosin; Pi, inorganic phosphate; RT‐qPCR, reverse transcription‐quantitative polymerase chain reaction; Runx2, Runt‐related transcription factor 2; SCr, serum creatinine; U‐pro, urine protein; VC, vascular calcification; VSMCs, vascular smooth muscle cells; α‐SMA, α‐smooth muscle actin. *P < .05 indicates significant difference. Data (mean ± SD) between two groups were analysed using unpaired *t* test. Data among multiple groups were compared by one‐way analysis of variance (ANOVA) with Tukey's post hoc test. The experiment was performed in triplicate

To further investigate the expression of HDAC1 in VC in CRF models, the rats were first fed with forage containing 0.75% adenine to establish a CRF model, and then supplemented with a high‐phosphorus diet to induce VC. We then examined the rat renal tissues with HE staining. The results depicted in Figure 1A indicate that the number of glomeruli was normal in the blank group, with no evidence of fibrosis and crescent lesions observed under the microscope. In addition, the tubular epithelium was intact with no signs of degeneration, necrosis and tube type, while there was no sign of inflammatory cell infiltration or fibrosis in the renal interstitium. However, in the CRF + VC group, the glomerular structure was distinctly disordered, with the renal tubules observed to be dilated and edematous, accompanied by inflammatory cell infiltration in the lumen, and brown crystalline deposition in the tubules. Additionally, rats in the CRF + VC group also showed notably diminished signs of interstitial fibrosis and renal vessels when compared to the normal rats. Subsequent analyses revealed elevated levels of SCr, BUN and U‐pro in the serum of rats in the CRF + VC group (Figure 1B‐D). Alizarin red staining of thoracic aorta revealed a normal vascular structure in the blank group, while there was a continuous linear distribution of calcified nodules in the middle membrane of the artery in the CRF + VC group (Figure 1E). Western blot analysis was conducted to detect the expression of osteogenic differentiation‐related transcription factors Runx2 and α‐SMA in aortic tissues. As depicted in Figure 1F, the expression of Runx2 was markedly higher, while that of α‐SMA was lower, in the CRF + VC group when compared to the blank group. These findings together indicated successful model establishment. Western blot analysis was subsequently employed to detect the expression of HDAC1 in thoracic aorta, which showed low levels of HDAC1 in the CRF + VC group relative to the blank group (Figure 1G). Next, to verify further the expression of HDAC1 in VC, the primary VSMCs of rats were treated with high Pi (2 mmol/L) in vitro, followed by von Kossa staining to evaluate their induced calcium deposition. The results in Figure 1H demonstrate notable signs of calcium deposition in VSMCs after high Pi induction. Western blot analysis revealed reduced expression of HDAC1 in the high Pi‐induced VSMCs (Figure 1I). Therefore, it was suggested that HDAC1 inhibition correlated with the formation of VC in vivo and in vitro.

### 3.2 The HDAC1 reduced the formation of VC in vivo and in vitro

**Figure 2:**
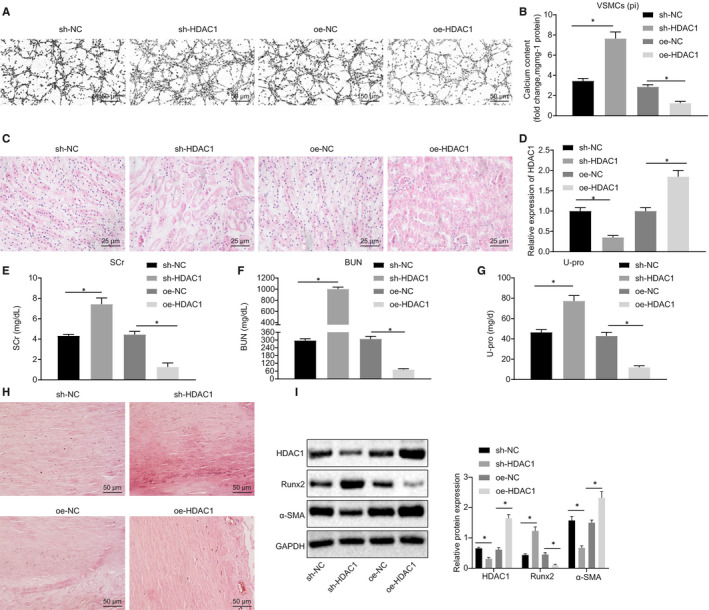
HDAC1 reduces the formation of VC in adenine‐induced CRF rats and high phosphate‐induced VSMCs. A, Von Kossa staining of calcium deposition in VSMCs (200×). B, Calcium content in cell supernatant measured using a colorimetric method. C, HE staining of renal tissues (400×). D, HDAC1 expression in rats determined using RT‐qPCR. E‐G, Serum levels of SCr, BUN and U‐pro measured by the Falcor 300 analyser. H, Calcification of thoracic aorta (200×) determined by Alizarin red staining. I, Western blot analysis of Runx2 and α‐SMA proteins in aortic tissues. BUN, blood urea nitrogen; CRF, chronic renal failure; GAPDH, glyceraldehyde‐3‐phosphate dehydrogenase; HDAC1, histone deacetylase 1; HE, haematoxylin‐eosin; NC, negative control; oe, overexpression; RT‐qPCR, reverse transcription‐quantitative polymerase chain reaction; Runx2, Runt‐related transcription factor 2; SCr, serum creatinine; sh, short hairpin; U‐pro, urine protein; VC, vascular calcification; VSMCs, vascular smooth muscle cells; α‐SMA, α‐smooth muscle actin. *P < .05 indicates significant difference. Data (mean ± SD) between two groups were analysed using unpaired *t* test. Data among multiple groups were compared by one‐way analysis of variance (ANOVA) with Tukey's post hoc test. The experiment was conducted in triplicate

Next, to evaluate further the mechanism of HDAC1 in VC of CRF, VSMCs induced by high Pi were transfected with sh‐HDAC1 or oe‐HDAC1 plasmids, and their calcium deposition was detected by von Kossa staining. As illustrated in Figure 2A, calcium deposition in VSMCs transfected with sh‐HDAC1 was significantly elevated while that in the VSMCs transfected with oe‐HDAC1 was significantly decreased. Colorimetric analysis of calcium content in the cell supernatant showed an increase in the VSMCs with absence of HDAC1, while reduced calcium level was identified in the VSMCs in the presence of HDAC1 (Figure 2B). To clarify further the role of HDAC1 in VC in CRF rats, the rats were injected with lentivirus‐packaged oe‐HDAC1 and sh‐HDAC1 plasmids, and their renal tissues were later analysed using HE staining. As depicted in Figure 2C, the rats treated with oe‐HDAC1 exhibited a greater number of glomeruli, diminished degree of tubular dilatation and decreased inflammatory cells and intraluminal crystallization of adenine. The expression of HDAC1 measured using RT‐qPCR and Western blot analysis elevated in the oe‐HDAC1‐treated rats, while decreased HDAC1 expression was observed in sh‐HDAC1‐treated rats (Figure 2D,I). The results further illustrated a decline in the serum levels of SCr, BUN and U‐pro in rats treated with oe‐HDAC1 (Figure 2E‐G). Alizarin red staining indicated a significant decrease in the calcium deposition in the thoracic aorta of the rats treated with oe‐HDAC1 (Figure 2H). Colorimetric assay showed that the calcium content was markedly reduced in the aortic samples from rats with oe‐HDAC1 (Figure 2I). Finally, Western blot analysis results demonstrated a notably decreased expression of Runx2 in response to oe‐HDAC1 treatment, while the expression of α‐SMA was up‐regulated in aortic tissues.

### 3.3 Autophagy was vasculoprotective against calcification in vivo and in vitro

**Figure 3:**
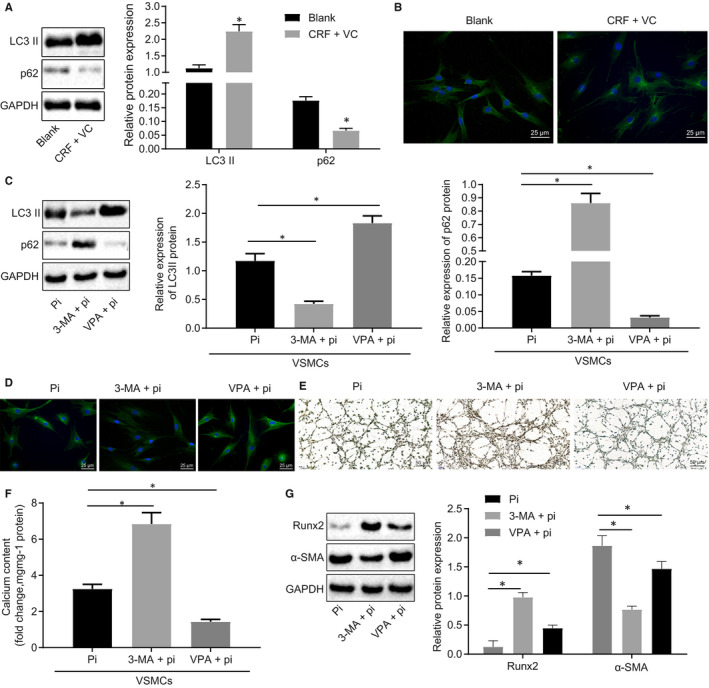
Autophagy confers vasculoprotective against calcification in adenine‐induced CRF rats and high phosphate‐induced VSMCs. A, Western blot analysis of LC3 II and p62 proteins in renal tissues. B, Immunofluorescence staining of LC3 II in renal tissues (400×). C, Western blot analysis of LC3 II and p62 proteins in VSMCs. D, Immunofluorescence staining of LC3 II in VSMCs (400×). E, Von Kossa staining of calcium deposition in VSMCs (200×). F, Calcium content in cell supernatant measured using a colorimetric method. G, Western blot analysis of Runx2 and α‐SMA proteins in VSMCs. BUN, blood urea nitrogen; CRF, chronic renal failure; GAPDH, glyceraldehyde‐3‐phosphate dehydrogenase; HDAC1, histone deacetylase 1; Pi, inorganic phosphate; Runx2, Runt‐related transcription factor 2; SCr, serum creatinine; U‐pro, urine protein; VC, vascular calcification; VPA, valproic acid; VSMCs, vascular smooth muscle cells; α‐SMA, α‐smooth muscle actin. *P < .05 indicates significant difference. Data (mean ± SD) between two groups were analysed using unpaired *t* test, while data among multiple groups were assessed using one‐way ANOVA with Tukey's post hoc test. The experiment was run in triplicate

Western blot analysis was performed to detect the protein expression of LC3 II and p62 in thoracic aortas, the results of which are depicted in Figure 3A. In the CRF + VC group, the expression of LC3 II was elevated, while that of p62 was reduced. The expression of LC3 II in aortic tissues was detected by immunofluorescence staining, which showed that the expression of LC3 II was up‐regulated in the CRF + VC group (Figure 3B). To investigate the potential role of autophagy in high Pi‐provoked VC in VSMCs, VSMCs were treated with autophagy inhibitor 3‐MA (5 mmol/L) and autophagy inducer VPA (1 mmol/L), respectively. Western blot analysis of the expression of LC3 II and p62 proteins in VSMCs (Figure 3C) showed that treatment with 3‐MA down‐regulated LC3 II expression, but yet up‐regulated p62 expression. However, opposite results were seen in VPA‐treated VSMCs. The immunofluorescence staining results demonstrated that the expression of LC3 II was decreased in response to 3‐MA treatment, but up‐regulated in response to VPA treatment (Figure 3D). Alizarin red staining analyses exhibited increased calcium deposition in 3‐MA‐treated cells, whereas there was significantly less calcium deposition in VPA‐treated cells (Figure 3E). Moreover, Figure 3F showed notably increased calcium content upon 3‐MA treatment, which was blocked by VPA treatment. Western blot analysis showed elevated Runx2 expression in the cells treated with 3‐MA, while there was a reduction in α‐SMA expression. Opposite effects on these factors were observed in the in cells treated with VPA (Figure 3G). These results suggested that autophagy inhibited vascular calcification in vivo and in vitro.

### 3.4 HDAC1 deacetylated LSD1 and thus mediated autophagy in VC in vitro

**Figure 4:**
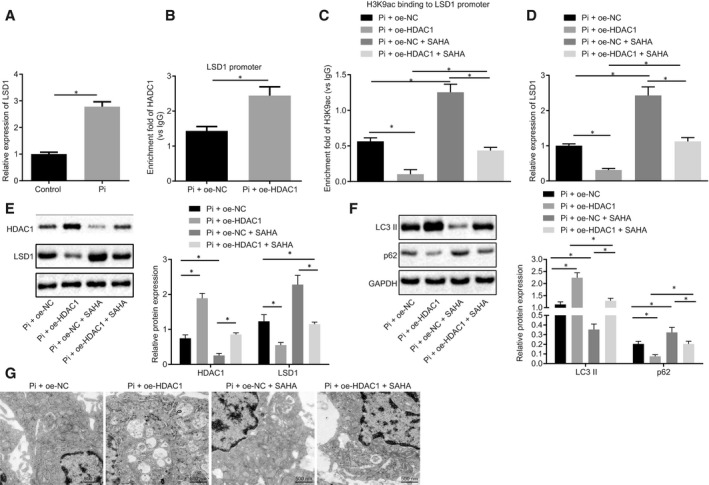
HDAC1 deacetylates LSD1 and thus mediates autophagy in high phosphate‐induced VC models. A, LSD1 expression in VSMCs without treatment or treated with high Pi detected using RT‐qPCR. B, The enrichment of HDAC1 in the promoter region of LSD1 measured using the ChIP assay. C, The enrichment of H3K9ac in the promoter region of LSD1 measured using ChIP assay. D, LSD1 expression in cells treated with oe‐HDAC1 or SAHA (inhibitor of HDAC1) detected using RT‐qPCR. E, Western blot analysis of LSD1 and HDAC1 protein in cells. F, Western blot analysis of LC3 II and p62 proteins in cells. G, The formation of autophagosomes observed under an electron microscope (20 000×). BUN, blood urea nitrogen; CRF, chronic renal failure; GAPDH, glyceraldehyde‐3‐phosphate dehydrogenase; HDAC1, histone deacetylase 1; HE, haematoxylin‐eosin; NC, negative control; oe, overexpression; Pi, inorganic phosphate; RT‐qPCR, reverse transcription‐quantitative polymerase chain reaction; Runx2, Runt‐related transcription factor 2; SCr, serum creatinine; U‐pro, urine protein; VC, vascular calcification; VPA, valproic acid; VSMCs, vascular smooth muscle cells; α‐SMA, α‐smooth muscle actin. *P < .05 indicates significant difference. Data (mean ± SD) between two groups were analysed using unpaired *t* test, while data among multiple groups were assessed using one‐way ANOVA with Tukey's post hoc test. The experiment was run in triplicate

RT‐qPCR analysis showed that LSD1 expression was significantly increased in VSMCs following high Pi induction (Figure 4A). Next, we used the ChIP to investigate the involvement of HDAC1 regulation on LSD1 acetylation in VSMCs induced by high Pi. Results showed increased HDAC1 enrichment in oe‐HDAC1‐treated cells (Figure 4B). Other ChIP assays showed reduced enrichment of H3K9ac in the LSD1 promoter region in response to oe‐HDAC1, while treatment with the HDAC1 inhibitor SAHA (10 μmol/L) led to elevated H3K9ac enrichment in the LSD1 promoter region (Figure 4C). Furthermore, RT‐qPCR and Western blot analysis showed that the mRNA and protein expression of HDAC1 was increased by oe‐HDAC1, and the mRNA and protein expression of LSD1 was inhibited by oe‐HDAC1, while treatment with SAHA had opposite effects (Figure 4D,E). The expression of LC3 II and p62 in cells following different treatments was detected by Western blot analysis, which revealed elevated LC3 II expression but reduced expression of p62 in cells with oe‐HDAC1 treatment. However, LC3 II expression was reduced, while p62 expression was elevated in SAHA‐treated cells, but these effects were abrogated following additional oe‐HDAC1 treatment (Figure 4F). The formation of autophagosomes was subsequently examined by electron microscopy, which revealed that the cells treated with oe‐HDAC1 had increased abundance of autophagosomes, while SAHA treatment led to a decrease in autophagosomes (Figure 4G). Based on these findings, we conclude that HDAC1 inhibits the expression of LSD1 by modifying the promoter region of LSD1 via H3K9ac, which consequently stimulates cell autophagy.

### 3.5 LSD1 reduced the expression of SESN2, thus inhibiting autophagy and promoting VC in vitro

**Figure 5:**
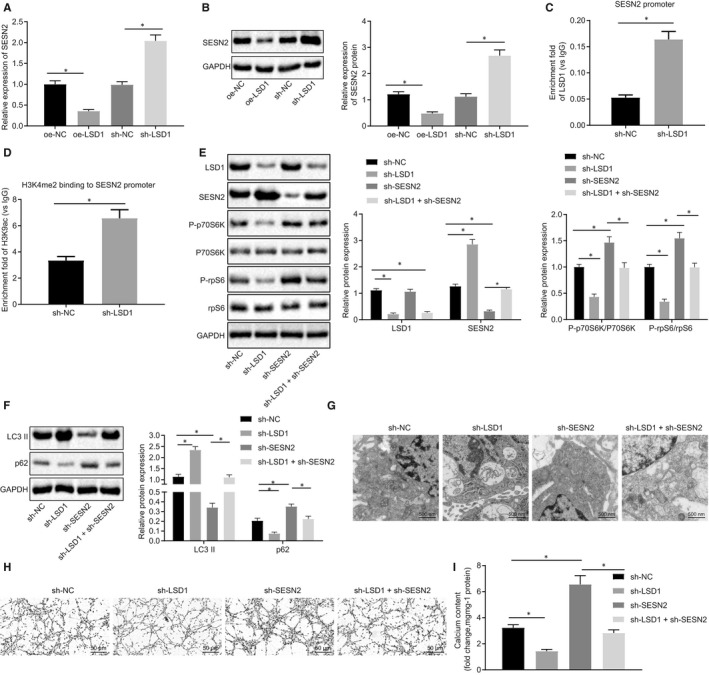
LSD1 inhibits autophagy and exacerbates VC by regulation of SESN2 demethylation in high phosphate‐induced VC model mice. A, SESN2 expression in VSMCs detected using RT‐qPCR. B, Western blot analysis of SESN2 protein in VSMCs. C, Enrichment of LSD1 in the promoter region of SESN2 measured by ChIP assay. D, Enrichment of H3K4me2 in the promoter region of SESN2 measured by ChIP assay. E, Western blot analysis of the mTOR signalling pathway‐related proteins. F, Western blot analysis of LC3 II and p62 protein in VSMCs. G, The formation of autophagosomes observed under an electron microscope (20 000×). H, Von Kossa staining of calcium deposition in VSMCs (200×). I, Calcium content in cell supernatant measured by colorimetric method. BUN, blood urea nitrogen; CRF, chronic renal failure; GAPDH, glyceraldehyde‐3‐phosphate dehydrogenase; HDAC1, histone deacetylase 1; HE, haematoxylin‐eosin; NC, negative control; oe, overexpression; RT‐qPCR, reverse transcription‐quantitative polymerase chain reaction; Runx2, Runt‐related transcription factor 2; SCr, serum creatinine; sh, short hair; U‐pro, urine protein; VC, vascular calcification; VPA, valproic acid; VSMCs, vascular smooth muscle cells; α‐SMA, α‐smooth muscle actin. *P < .05 indicates significant difference. Data (mean ± SD) between two groups were analysed using unpaired *t* test, while data among multiple groups were assessed using one‐way ANOVA with Tukey's post hoc test. The experiment was performed in triplicate

Next, to elucidate whether LSD1 affects autophagy through regulating SESN2 in high Pi‐induced VSMCs, we overexpressed or knocked down LSD1 in high Pi‐induced VSMCs. RT‐qPCR and Western blot analysis (Figure 5A,B) detected elevated SESN2 expression in response to sh‐LSD1 treatment, but reduced SESN2 expression following oe‐LSD1 treatment, suggesting that LSD1 could indeed regulate the expression of SESN2. ChIP assay to detect the enrichment of LSD1 in the promoter region of SESN2 (Figure 5C) demonstrated increased enrichment of LSD1 in oe‐LSD1‐treated cells. ChIP assay of H3K4me2 expression in the promoter region of SESN2 (Figure 5D) revealed elevated H3K4me2 expression upon sh‐LSD1 treatment. These results suggest that LSD1 could inhibit SESN2 expression through the demethylation of H3K4me2.

Next, to elucidate the effect of LSD1 regulation of SESN2 on the autophagy of high Pi‐induced VSMCs, we performed Western blot analysis to detect the expression of LSD1, SESN2 and mTOR signalling pathway‐related proteins, including phosphorylated p70S6K, p70S6K, phosphorylated rpS6 and rpS6. As illustrated in Figure 5E, sh‐LSD1‐treated cells showed decreased expression of LSD1 and phosphorylated p70S6K/p70S6K and phosphorylated rpS6/rpS6, yet up‐regulated SESN2 expression. On the other hand, opposite effects were identified in the sh‐SESN2‐treated cells. The Western blot analysis results demonstrated that the expression of LC3 II was promoted, while p62 expression was inhibited in sh‐LSD1‐treated cells, which was completely reversed by sh‐SESN2 treatment (Figure 5F). Electron microscopy showed that the cells treated with sh‐LSD1 had increased autophagosome density, while sh‐SESN2 treatment led to reduced autophagosomes (Figure 5G). Alizarin red staining showed a reduced calcium deposition in sh‐LSD1‐treated cells, whereas increased calcium deposition was observed in sh‐SESN2‐treated cells (Figure 5H). Colorimetric analysis of cell supernatants showed reduced calcium content following sh‐LSD1 treatment, whereas sh‐SESN2 treatment resulted in increased calcium content (Figure 5I). These results constituted evidence that LSD1 could inhibit autophagy and may potentially contribute to deteriorated VC by regulating demethylation of SESN2.

### 3.6 The novel molecular pathway of HDAC1/LSD1/SESN2 was validated in CRF‐induced VC

**Figure 6:**
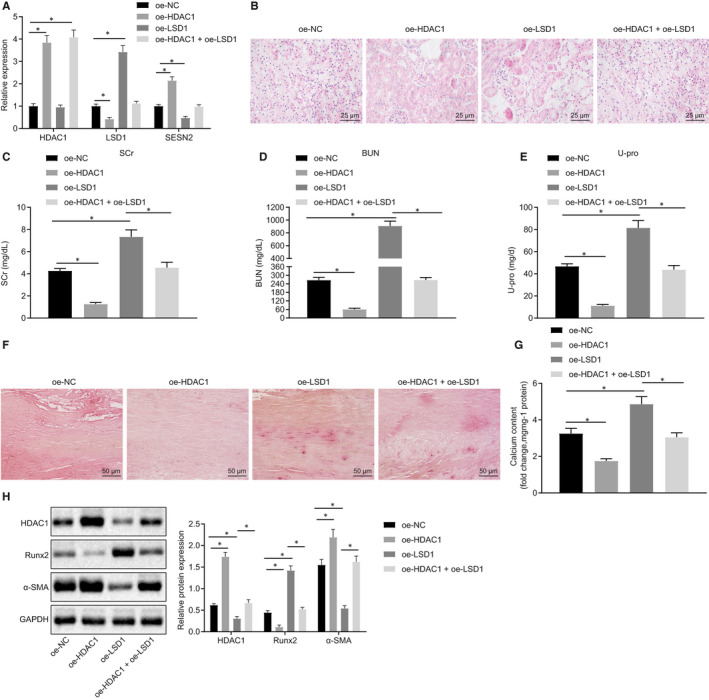
Regulation of the HDAC1/LSD1/SESN2 pathway on VC in adenine‐induced CRF rats. A, The expression of HDAC1, LSD1 and SESN2 in thoracic aorta of rats determined using RT‐qPCR. B, HE staining of pathological changes of renal tissues (400×). C‐E, Serum levels of SCr, BUN, and U‐pro in rats measured by automated analyser Falcor 300. F, Alizarin red staining of calcification of thoracic aorta (200×). G, Calcium content in aortic tissue supernatant measured by colorimetric method. H, Western blot analysis of Runx2 and α‐SMA proteins in aortic tissues. BUN, blood urea nitrogen; CRF, chronic renal failure; GAPDH, glyceraldehyde‐3‐phosphate dehydrogenase; HDAC1, histone deacetylase 1; HE, haematoxylin‐eosin; NC, negative control; oe, overexpression; Pi, inorganic phosphate; RT‐qPCR, reverse transcription‐quantitative polymerase chain reaction; Runx2, Runt‐related transcription factor 2; SCr, serum creatinine; U‐pro, urine protein; VC, vascular calcification; VPA, valproic acid; VSMCs, vascular smooth muscle cells; α‐SMA, α‐smooth muscle actin. *P < .05 indicates significant difference. Data (mean ± SD) between two groups were analysed using unpaired *t* test. The experiment was run in triplicate

We have seen that the HDAC1/LSD1/SESN2 pathway could regulate the calcification of VSMCs induced by high Pi by affecting autophagy. To further verify this finding in vivo, rats with CRF were treated with lentiviruses, and effects on HDAC1, LSD1 and SESN2 expression were subsequently detected by RT‐qPCR. The results revealed up‐regulated HDAC1 and SESN2, but down‐regulated LSD1 in aortic tissues of rats treated with oe‐HDAC1 (Figure 6A). In contrast, the oe‐LSD1‐treated rats exhibited no notable changes in HDAC1 expression, but did show elevated expression of LSD1 and reduced expression of SESN2. The rats treated with oe‐HDAC1 + oe‐LSD1 showed increased HDAC1 expression but no notable changes in the expression of LSD1 and SESN2. Pathological examination of renal tissue with HE staining (Figure 6B) showed that rats treated with oe‐HDAC1 exhibited a greater number of glomeruli, reduced degree of tubular dilatation and decreased presence of inflammatory cells and crystallization of adenine. In contrast, the rats treated with oe‐LSD1 exhibited opposite effects on these pathological markers. The subsequent results demonstrated reduced serum levels of SCr, BUN and U‐pro in rats treated with oe‐HDAC1, which could be reversed by oe‐LSD1 treatment (Figure 6C‐E). Alizarin red staining of the rat thoracic aorta showed reduced calcium deposition upon treatment with oe‐HDAC1, but increased calcium deposition with oe‐LSD1 treatment (Figure 6F). Colorimetric analysis revealed a notable reduction in calcium content detected in rats treated with oe‐HDAC1, while increased calcium content was detected following oe‐LSD1 treatment (Figure 6G). Finally, Western blot analysis of aortic tissues showed notably diminished expression of Runx2 upon oe‐HDAC1 treatment, while α‐SMA expression was increased significantly, whereas opposite effects were seen following oe‐LSD1 treatment (Figure 6H). These results suggested that the HDAC1/LSD1/SESN2 pathway likely regulated the VC process in rats with CRF.

## 4 DISCUSSION

Chronic renal failure is a severe clinical disorder, and widely considered to be the final manifestation of a wide variety of chronic kidney diseases.^19^ Emerging evidence documented that the accumulation of toxins and water retention occurs as a result of renal dysfunction on CRF.^1^ Patients suffering from CRF have a distinctly elevated risk of developing cardiovascular diseases.^20^ VC as a result of increased Pi levels has been highlighted as the leading contributor to cardiovascular dysfunction following CRF.^21^ Thus, identifying biomarkers for VC may help in predicting the progression of the disease and its cardiovascular manifestations. The current study was designed with the aim to investigate the potential mechanism by which HDAC1 influences the progression of VC after CRF. Present in vivo and in vitro findings indicated that up‐regulation of HDAC1 could prevent the onset of VC after CRF via impeding LSD1 expression and blockade of the SESN2‐dependent mTOR signalling pathway.

A key initial finding of our study was that renal HDAC1 expression was reduced in rats with CRF in vivo and in vitro. Low HDAC1 expression has been reported in acute kidney injury in vitro, and the unhindered activity of HDAC1 has been regarded as a prerequisite factor for renal protection and regeneration following acute kidney injury.^8^ The deletion of the HDAC1 gene from the ureteric bud cell lineage in mice leads to bilateral renal hypodysplasia.^22^ Moreover, diminished HDAC1 expression has been frequently reported in studies of kidney ischemia‐ and reperfusion‐induced injury.^23^ Our results also demonstrated that HDAC1 could suppress VC in CRF both in vivo and in vitro. Previous literature has indicated VC to be the predominant contributor to cardiovascular dysfunction in CRF.^24^ Interestingly, HDAC activity has been identified as a potential therapeutic target in VC due to its active post‐translational modification.^25^ The deletion of HDAC1 activity via genetic ablation or pharmacological inhibition is capable of augmenting the severity of VC.^10^ Thus, enhanced HDAC1 expression could potentially attenuate VC following CRF.

An additional crucial finding of the current study was our observation that promotion of autophagy could inhibit VC in CRF. Autophagy has been described as a homoeostatic mechanism whereby proteins and organelles are detached and subsequently recycled into a general metabolic pool.^26^ Furthermore, autophagy has been regarded as a promising endogenous protective mechanism against Pi‐induced VC via its effects on the reduction in matrix vesicle release.^27^ Existing literature has suggested that HDAC1 is closely associated with the process of autophagy in mice owing to its ability to regulate skeletal muscle homoeostasis and autophagy flux.^11^ Moreover, HDAC1 has been shown to bind to the promoter region of LSD1 and subsequently mediate the deacetylation of LSD1, which consequently affects the expression of LSD1.^13^ Notably, the inhibition of LSD1, either by small interference RNA (siRNA) or pharmacological agents, provokes the activation of autophagy in various gynaecologic malignancies.^14^ Consistent with the present findings, a previous report concluded that HDAC1 possesses the ability to inhibit the expression of LSD1 by modifying the promoter region of LSD1 via H3K9ac, thereby stimulating cell autophagy.

The current study also revealed that LSD1 could restrain autophagy and potentiate VC via the regulation of SESN2 demethylation in CRF. Previous research has documented that silencing of LSD1 triggers autophagy in ovarian cancer cells via the AKT/mTOR signalling pathway.^28^ LSD1 has been reported to bind to the promoter region of SESN2, a critical regulator of mTORC1 activity, while the inhibition of LSD1 may stimulate SESN2 expression, which can disrupt mTORC1 activity and ultimately enhance autophagy.^29^ A trend towards increased expression of SESN2 upon LSD1 inhibition was identified during our study, but the expression of SESN2 was markedly reduced in VSMCs following oe‐LSD1 treatment. In addition, elevated H3K4me2 expression was observed upon sh‐LSD1 treatment. These findings suggest that LSD1 may inhibit SESN2 expression through the demethylation of H3K4me2. Moreover, our in vivo experimental results also demonstrated that the HDAC1/LSD1/SESN2 axis may potentially regulate VC process in rats with CRF.

**Figure 7:**
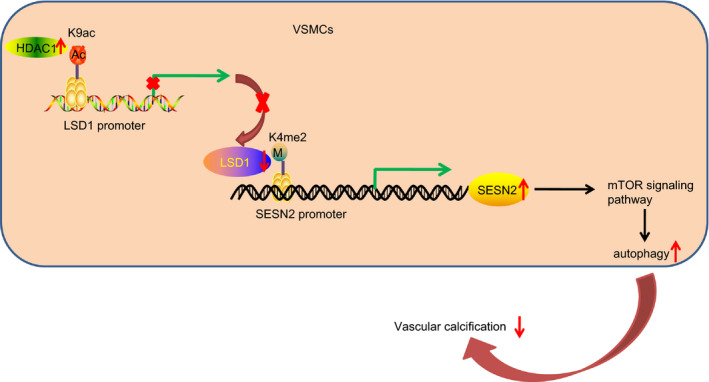
A mechanistic map depicting the potential role of the HDAC1/LSD1/SESN2 pathway in VC in CRF. HDAC1 regulates the deacetylation of LSD1 promoter region through H3K9ac and then inhibits the expression of LSD1. After the inhibition of LSD1, H3K4me2 binds into the promoter region of SESN2 to promote the expression of SESN2. SESN2 promotes autophagy of VSMCs via mTOR signalling pathway regulation which ultimately alleviated VC in CRF. BUN, blood urea nitrogen; CRF, chronic renal failure; HDAC1, histone deacetylase 1; Runx2, Runt‐related transcription factor 2; SCr, serum creatinine; U‐pro, urine protein; VC, vascular calcification; VPA, valproic acid; VSMCs, vascular smooth muscle cells; α‐SMA, α‐smooth muscle actin

In conclusion, the in vitro and in vivo results of the current study provide consistent evidence demonstrating that the overexpression of histone deacetylase HDAC1 potentially impedes the onset of VC in CRF via LSD1 inhibition through the SESN2‐dependent mTOR signalling pathway (Figure 7). These results provide novel insight into the inhibition of HDAC1‐mediated LSD1 as a promising competitive new target in VC after CRF. However, there is a need to confirm present results in animal and cell models by analysis of the HDAC1/LSD1/SESN2 pathway in tissue specimens from CRF‐diagnosed patients.

## CONFLICT OF INTEREST

The author declares no competing interest exists.

## AUTHOR CONTRIBUTION

**Jiajun Zhou:** Conceptualization (equal); Resources (equal). **Han Zhou:** Data curation (equal); Software (equal). **Caixin Liu:** Investigation (equal); Methodology (equal); Project administration (equal). **Lin Huang:** Validation (equal); Writing‐review & editing (equal). **Dongmei Lu:** Formal analysis (equal); Supervision (equal). **Chaoqing Gao:** Visualization (equal); Writing‐original draft (equal).

## Data Availability

Research data not shared.

## References

[1] Vadakedath S , Kandi V . Dialysis: a review of the mechanisms underlying complications in the management of chronic renal failure. Cureus. 2017;9:e1603. 29067226 10.7759/cureus.1603 PMC5654453

[2] Patel MV , Gupta SN , Patel NG . Effects of ayurvedic treatment on 100 patients of chronic renal failure (other than diabetic nephropathy). AYU. 2011;32:483‐486. 22661841 10.4103/0974-8520.96120 PMC3361922

[3] Norman JT , Fine LG . Intrarenal oxygenation in chronic renal failure. Clin Exp Pharmacol Physiol. 2006;33:989‐996. 17002678 10.1111/j.1440-1681.2006.04476.x

[4] Humphris JL , Patch AM , Nones K , et al. Hypermutation in pancreatic cancer. Gastroenterology. 2017;152:68‐74 e2. 27856273 10.1053/j.gastro.2016.09.060

[5] Lian Y , Xie L , Chen M , Chen L . Effects of an astragalus polysaccharide and rhein combination on apoptosis in rats with chronic renal failure. Evid Based Complement Alternat Med. 2014;2014:271862. 24711851 10.1155/2014/271862 PMC3966320

[6] Kaluza D , Kroll J , Gesierich S , et al. Histone deacetylase 9 promotes angiogenesis by targeting the antiangiogenic microRNA‐17‐92 cluster in endothelial cells. Arterioscler Thromb Vasc Biol. 2013;33:533‐543. 23288173 10.1161/ATVBAHA.112.300415

[7] Liu N , Zhuang S . Treatment of chronic kidney diseases with histone deacetylase inhibitors. Front Physiol. 2015;6:121. 25972812 10.3389/fphys.2015.00121 PMC4411966

[8] Tang J , Yan Y , Zhao TC , et al. Class I HDAC activity is required for renal protection and regeneration after acute kidney injury. Am J Physiol Renal Physiol. 2014;307:F303‐F316. 24808536 10.1152/ajprenal.00102.2014 PMC4121572

[9] Kiweler N , Brill B , Wirth M , et al. The histone deacetylases HDAC1 and HDAC2 are required for the growth and survival of renal carcinoma cells. Arch Toxicol. 2018;92:2227‐2243. 29845424 10.1007/s00204-018-2229-5

[10] Kwon D‐H , Eom GH , Ko JH , et al. MDM2 E3 ligase‐mediated ubiquitination and degradation of HDAC1 in vascular calcification. Nat Commun. 2016;7:10492. 26832969 10.1038/ncomms10492 PMC4740400

[11] Moresi V , Carrer M , Grueter CE , et al. Histone deacetylases 1 and 2 regulate autophagy flux and skeletal muscle homeostasis in mice. Proc Natl Acad Sci USA. 2012;109:1649‐1654. 22307625 10.1073/pnas.1121159109 PMC3277131

[12] Qi YY , Zhou XJ , Zhang H . Autophagy and immunological aberrations in systemic lupus erythematosus. Eur J Immunol. 2019;49:523‐533. 30776086 10.1002/eji.201847679

[13] Nalawansha DA , Pflum MK . LSD1 substrate binding and gene expression are affected by HDAC1‐mediated deacetylation. ACS Chem Biol. 2017;12:254‐264. 27977115 10.1021/acschembio.6b00776 PMC5490840

[14] Chao A , Lin C‐Y , Chao A‐N , et al. Lysine‐specific demethylase 1 (LSD1) destabilizes p62 and inhibits autophagy in gynecologic malignancies. Oncotarget. 2017;8:74434‐74450. 29088798 10.18632/oncotarget.20158 PMC5650353

[15] Wang Z , Long Q‐Y , Chen L , et al. Inhibition of H3K4 demethylation induces autophagy in cancer cell lines. Biochim Biophys Acta Mol Cell Res. 2017;1864:2428‐2437. 28800922 10.1016/j.bbamcr.2017.08.005

[16] Wang Z , Zhu Q , Li PL , et al. Silencing of hypoxia‐inducible factor‐1alpha gene attenuates chronic ischemic renal injury in two‐kidney, one‐clip rats. Am J Physiol Renal Physiol. 2014;306:F1236‐F1242. 24623146 10.1152/ajprenal.00673.2013 PMC4024731

[17] Huang D‐N , Lu C‐Y , Shao P‐L , et al. In vivo inhibition of influenza A virus replication by RNA interference targeting the PB2 subunit via intratracheal delivery. PLoS One. 2017;12:e0174523. 28380007 10.1371/journal.pone.0174523 PMC5381882

[18] Cai Y , Wang XL , Flores AM , Lin T , Guzman RJ . Inhibition of endo‐lysosomal function exacerbates vascular calcification. Sci Rep. 2018;8:3377. 29467541 10.1038/s41598-017-17540-6 PMC5821871

[19] Zhao YY , Liu J , Cheng XL , Bai X , Lin RC . Urinary metabonomics study on biochemical changes in an experimental model of chronic renal failure by adenine based on UPLC Q‐TOF/MS. Clin Chim Acta. 2012;413:642‐649. 22227165 10.1016/j.cca.2011.12.014

[20] Faure V , Dou L , Sabatier F , et al. Elevation of circulating endothelial microparticles in patients with chronic renal failure. J Thromb Haemost. 2006;4:566‐573. 16405517 10.1111/j.1538-7836.2005.01780.x

[21] Manivannan J , Barathkumar TR , Sivasubramanian J , et al. Diosgenin attenuates vascular calcification in chronic renal failure rats. Mol Cell Biochem. 2013;378:9‐18. 23423339 10.1007/s11010-013-1588-8

[22] Chen S , Yao X , Li Y , et al. Histone deacetylase 1 and 2 regulate Wnt and p53 pathways in the ureteric bud epithelium. Development. 2015;142:1180‐1192. 25758227 10.1242/dev.113506 PMC4360175

[23] Kim JI , Jung KJ , Jang HS , Park KM . Gender‐specific role of HDAC11 in kidney ischemia‐ and reperfusion‐induced PAI‐1 expression and injury. Am J Physiol Renal Physiol. 2013;305:F61‐F70. 23657855 10.1152/ajprenal.00015.2013

[24] Ou Y , Liu Z , Li S , et al. Citrate attenuates vascular calcification in chronic renal failure rats. APMIS. 2017;125:452‐458. 28332248 10.1111/apm.12667

[25] Kwon DH , Kim YK , Kook H . New aspects of vascular calcification: histone deacetylases and beyond. J Korean Med Sci. 2017;32:1738‐1748. 28960024 10.3346/jkms.2017.32.11.1738 PMC5639052

[26] Satriano J , Sharma K . Autophagy and metabolic changes in obesity‐related chronic kidney disease. Nephrol Dial Transplant. 2013;28(Suppl 4):iv29‐iv36. 23901047 10.1093/ndt/gft229 PMC3814227

[27] Dai X‐Y , Zhao M‐M , Cai Y , et al. Phosphate‐induced autophagy counteracts vascular calcification by reducing matrix vesicle release. Kidney Int. 2013;83:1042‐1051. 23364520 10.1038/ki.2012.482

[28] Feng S , Jin Y , Cui M , Zheng J . Lysine‐specific demethylase 1 (LSD1) inhibitor S2101 induces autophagy via the AKT/mTOR pathway in SKOV3 ovarian cancer cells. Med Sci Monit. 2016;22:4742‐4748. 27914215 10.12659/MSM.898825 PMC5142589

[29] Ambrosio S , Saccà CD , Amente S , et al. Lysine‐specific demethylase LSD1 regulates autophagy in neuroblastoma through SESN2‐dependent pathway. Oncogene. 2017;36:6701‐6711. 28783174 10.1038/onc.2017.267 PMC5717079

